# Supplementary material for: Exploration of Mental Readiness for Enhancing Dentistry in an Inter-Professional Climate
Source: Int J Environ Res Public Health. 2021 Jul 1;18(13):7038. doi: 10.3390/ijerph18137038 (PMC8297289; doi:10.3390/ijerph18137038)
Supplement: Supplementary file 1 [file ijerph-18-07038-s001.zip › ijerph-1277406-SI.pdf]

SUPPLEMENTARY MATERIALS
S1-S3

Exploration of Mental Readiness for Enhancing Dentistry in an Inter-Professional Climate

Judy McDonald[1] and Corrado Paganelli[2]

Supplementary Materials S1:

Personal Profile of Challenging Situations in Dentistry

Knowing your strengths and limitations can boost your self-confidence. Review the list of challenges below identified by seasoned dentists (add to each category as needed). For each challenging situation, decide and mark as follows:

- ~~Stroke out~~ if it is not applicable
- Circle if you would find it difficult to handle
- <u>Underline</u> if you generally feel comfortable handling it

This personal profile of challenging situations can assist you in realizing new strategies and refining your existing ones. Some situations may remain a limitation and will require a referral. Situations you feel comfortable and competent in handling may assist you in finding opportunities to mentor others and discovering possible areas for specialization.

Challenging Situations for Dentists
**Particular patient behaviour:** uneducated individuals · unbelievably picky patient's husband · distracting patient behaviour · dishonesty (e.g., not disclosing high b.p.) · ensure not alone with patient especially male-female · uncomfortable patient · patient who moves in the chair · very poor oral hygiene–not doing their part · cannot afford proper treatment due to low-income–knowing the treatment they can afford is not going to last · undergoing treatment with certain medications (e.g., bisphosphonate causing certain jaw damage) · cancer patients with really damaged white blood cells · patients with angina or cardiac conditions · patient might not tell you everything–not knowing signs versus symptoms · those really afraid of the needle · people with the attitude: *"It's my money. I'm paying you so you will do what I want."* · people feeling faint from the anesthetic · a high-risk patient is an uncomfortable patient · poor motor skills to perform a hand-skill to clean mouth or just don't have good health · patients who don't want to do anything unless the insurance will pay · patients experiencing homelessness (a love-hate relationship)–people destroy their lives and their teeth on drugs, methyl ice, crack, cocaine · children are definitely one of the hard ones–a crying and kicking child is not necessarily in pain.
**Degree of difficulty/risk:** extraction of third molars · bone grafts · implants · torn tongue · ankylosed tooth (risk of soldiered to bone) · being tired at end of the day · lawsuits are the number one fear of dentists · relieving pain after the patient leaves · complications · not knowing how to deal with the possible complications for a procedure · a simple ortho (or braces) treatment · possibility of permanent damage to nerves from extractions of impacted molars, root canals (e.g., in a molar) –anything where you might permanently lose the tooth · matching the colour and angulations on two front teeth · suddenly realize the tooth you're working on should've had a root canal · higher the level of dentistry, the more costly the service for the dentist · root canal in a molar · looking for the orifices or the entrances of the canals (especially if they are classified) · getting the right vertical dimensions · countries where there are not enough oral surgeons and taking the roots off becomes your responsibility.
**Degree of complexity:** minor things are not hard but complex–opening flap, surgical extraction of teeth · mouth full of blood (need to consider if patient is in pain) · handle sutures for closing flip · ensuring no complications · patient has to be careful after freezing · extraction: : suddenly find certain teeth enclosed with bone (often guessing in some countries that do not use x-rays) · endodontics (root-canal treatment): lots of complications because of blockage of canal, internal shape of canal, resorption · sustain proper sterilization practices · equip clinic with update first aid · waste hazard disposal · lack of pharmacological knowledge · full-mouth rehab · surgery · everything is challenging—even the simplest procedures · Not having the person frozen when you're doing a procedure and you start to hurt them · everything is challenging—even the simplest procedures · relieving pain from patients · adding insurance to the equation · asking for money when they are not happy or satisfied
**Teaching/managing responsibilities:** staff issues (i.e., lateness, illness, computers, staff-client problem) · ensuring proper staff with basic CPR-First Aid · business part (versus just the technique) is huge! · asking for money if client is unhappy · dealing with insurance telling you what to do · having balance in my life.
**Special relationship pressures:** relatives or a family member ('relativeoma') or friends ('privatitis') · not having patience with kids and paedodontics · by law should not treat partners · multiple relationships to manage (dentist and staff/client/patient) · the private-space thing for some dentists · you start to hurt the patient from not having the person frozen when doing a procedure–I hate hurting people.

Supplementary Materials S2:

Operational Readiness Performance Indicators for Dentistry

Instructions for use: These 13 performance indicators were specifically designed to approximate the ratio of job-specific competencies for dentistry found in this study. That is, in addition to developing the technical (31%) and physical (23%) readiness skills required for operational readiness, the necessary isolation and emphasis are placed on developing mental readiness skills (46%)—that will ultimately make the difference between satisfactory and peak performance. Traditional technical and physical readiness skills are covered by the first seven indicators, and mental readiness skills by the remaining six—which reflects what we now know about the weight of each (see Figure 2).

It is important to understand that performance indicators and practices do not need to be equally reflected in the number of lectures, proportion of training or all supplemental assessments. However, in the final assessment of a "fully functioning dentist," trainees must demonstrate comprehension and competency (pass/fail) in all physical, technical and mental performance indicators. A "Pass" represents not only what has been studied but how trainees combine this knowledge with their own "soft-skill" ways in performing. This circumvents graduating with the required percentage of knowledge and procedures yet failing without the mental competencies to succeed. While more time will be spent after graduation on honing a specialty, a foundation in mental preparedness will provide the resiliency and sustainability for facing the inherent complexities and difficulties of the job.

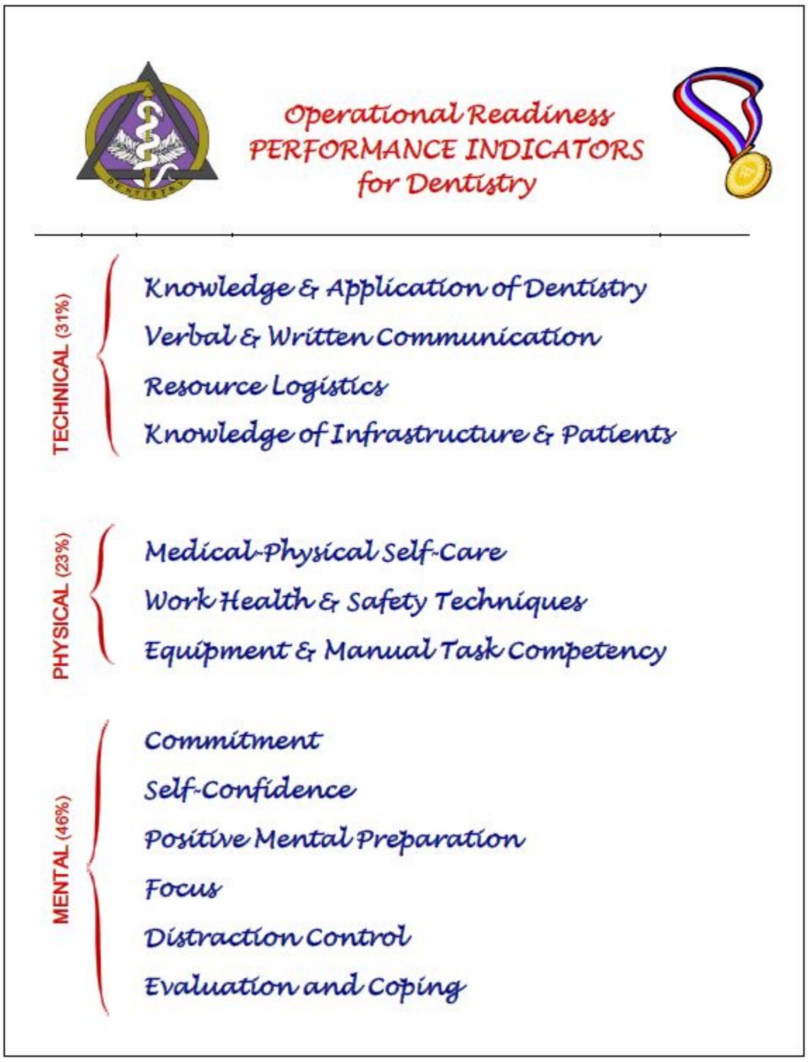

Supplementary Materials S3:

Clinical Training Assessment for Dentistry Trainees

Instructions for use: Instructors first familiarize their trainees up front with the expectations of clinical training and the goal of developing the 13 indicators of performance excellence. Consistent with the principles of adult education, trainees are expected to take an active role in their training by maintaining a daily log noting particular instances where they have demonstrated proficiency in the required 13 performance indicators. Clinical training can be divided into roughly three equal stages. At the end of each stage a progress report is completed by the instructor where the trainee is assessed (using the same form three times). The benchmark for the first progress report is the trainee's understanding of the indicators of performance excellence (comprehension). By the second report, trainees are assessed on their ability to demonstrate a growing competency in the indicators, and by the final report full competence is expected in all 13 indicators. If an instructor identifies a problem at any stage, it is discussed so the trainee can be given the opportunity to improve their performance. To ensure objectivity, instructors should back up their assessment with examples (or lack of examples) from the trainee's and their daily log.

CLINICAL TRAINING ASSESSMENT for Dentistry Trainees — **Progress Report** *(1 of 3)*

Trainee: ____________________ **Instructor/Mentor:** ____________________ **Training period dates:** ______________

Performance Indicators (Outcome)	Practices (that demonstrate outcome)	Daily Examples (Trainee is to assist in providing examples)
**Knowledge & Application of Dentistry**<br>Y N<br>☐ ☐ Comprehension<br>☐ ☐ Progressing competency<br>☐ ☐ Competent	knows and applies related dentistry education; has acquired expertise (e.g., materials, diagnosis, etc.); knows relevant local, international directives/laws (e.g., Food and Drug Administration, EU Directives); understands treatment and harm reduction; can analyze situations, problem solve and apply proper procedures; knows and applies related business practices; maintains necessary updates	
**Verbal & Written Communication**<br>Y N<br>☐ ☐ Comprehension<br>☐ ☐ Progressing competency<br>☐ ☐ Competent	is patient-directed; builds rapport, confidentiality; manages expectations; manages patient records (e.g., note taking, consent forms, clinical assessment); clear articulation of procedures; manages software and information technology	
**Resource Logistics**<br>Y N<br>☐ ☐ Comprehension<br>☐ ☐ Progressing competency<br>☐ ☐ Competent	identifies needs and goals; designs and coordinates patient treatment plan; schedules appointments efficiently; coordinates external services; accesses supplies; gains internal and external support; implements complex tasks; integrates flow of information/tasks; accesses resources and funds	
**Knowledge of Infrastructure & Patients**<br>Y N<br>☐ ☐ Comprehension<br>☐ ☐ Progressing competency<br>☐ ☐ Competent	knows dental practice or group structure ; knows physical layout of work area; follows local and international directives and procedures; knows and respects patient rights; knows community resources and patient referrals (e.g., hospitals, specialists)	
**Medical-Physical Self-Care**<br>Y N<br>☐ ☐ Comprehension<br>☐ ☐ Progressing competency<br>☐ ☐ Competent	has physical health and proper ergonomic positioning; manages fatigue (e.g., controls overbooking, takes breaks, proper rest); has control on dependence and addiction; is physically fit for the job (cardio, general stamina); has necessary injury recovery (back, neck, shoulder, etc.); eats and hydrates	
**Work Health & Safety Techniques**<br>Y N<br>☐ ☐ Comprehension<br>☐ ☐ Progressing competency<br>☐ ☐ Competent	is aware of personal safety (e.g., personal protective equipment, communicable disease awareness, disposal of contaminated objects, universal precautions); assesses environmental risks; ensures team compliance to safety measures	

Trainee: ____________________ **Instructor/Mentor:** ____________________ **Progress Report** *(2 of 3)*

Performance Indicators (Outcome)	Practices (that demonstrate outcome)	Examples from Daily Log (Trainee is to assist in providing examples)
**Equipment & Manual Task Competency**<br>Y N<br>☐ ☐ Comprehension<br>☐ ☐ Progressing competency<br>☐ ☐ Competent	effectively operates tools and instruments for the job (e.g., turbine, ultrasound, etc.); handles physical demands (e.g., correct chair positioning, standing, balancing, holding, controlling noise and vibrations); has equipment dexterity, coordination, accuracy and finesse; handles complex maneuvers and multitasking	
**Commitment**<br>Y N<br>☐ ☐ Comprehension<br>☐ ☐ Progressing competency<br>☐ ☐ Competent	has compassion to reduce pain; sets high standards; enjoys and is passionate about the work; persists through complexity; has integrity and a sense of responsibility for patients; remains sincere to avoid complaints and lawsuits; creates life-balance	
**Self-Confidence**<br>Y N<br>☐ ☐ Comprehension<br>☐ ☐ Progressing competency<br>☐ ☐ Competent	gains from past experience and training; assesses and accepts like-minded patients; knows and empowers patients; promotes team pride; refers to specialists; finds support in study groups and mentors; pursues continuous education (near and abroad)	
**Positive Mental Preparedness**<br>Y N<br>☐ ☐ Comprehension<br>☐ ☐ Progressing competency<br>☐ ☐ Competent	visualizes the final result; draws/rehearses with visual aids; pre-plans the logistics; allows ample time for procedures; has daily preparation rituals; treats patients with an optimistic attitude; develops a positive patient-relationship; explains plan or presents alternatives to waiting patients; prepares for the unexpected	
**Focus**<br>Y N<br>☐ ☐ Comprehension<br>☐ ☐ Progressing competency<br>☐ ☐ Competent	has total concentration and comfort amidst distractions; communicates non-verbally; stays calm and relaxed; has a patient-centred mind-set; is nice; feels rhythm and flow in the procedure; creates effortlessness in the practice	
**Distraction Control**<br>Y N<br>☐ ☐ Comprehension<br>☐ ☐ Progressing competency<br>☐ ☐ Competent	can immediately control excitement; is honest with patients; is willing to apologize, be flexible, redo and/or correct; puts distractions on hold; takes charge and push through; knows when to refer; instills calmness; takes breaks as needed to refocus	
**Evaluation and Coping**<br>Y N<br>☐ ☐ Comprehension<br>☐ ☐ Progressing competency<br>☐ ☐ Competent	uses self-reflection; sets realistic goals; seeks feedback (e.g., study group, patients, continuing education); recognizes and assesses technological changes; does the best possible; adjusts after an error; copes with unexpected setbacks; practices work-life balance	

Progress Report *(3 of 3)*

Trainee Comments/Improvement Strategies:

Print name: ____________________ Signature: ____________________ Date: ____________

Instructor/Mentor's Comments/Improvement Strategies:

Print name: ____________________ Signature: ____________________ Date: ____________
